# Supplementary figures and images for: Prophages integrating into prophages: A mechanism to accumulate type III secretion effector genes and duplicate Shiga toxin-encoding prophages in Escherichia coli
Source: PLoS Pathog. 2021 Apr 29;17(4):e1009073. doi: 10.1371/journal.ppat.1009073 (PMC8112680; doi:10.1371/journal.ppat.1009073)

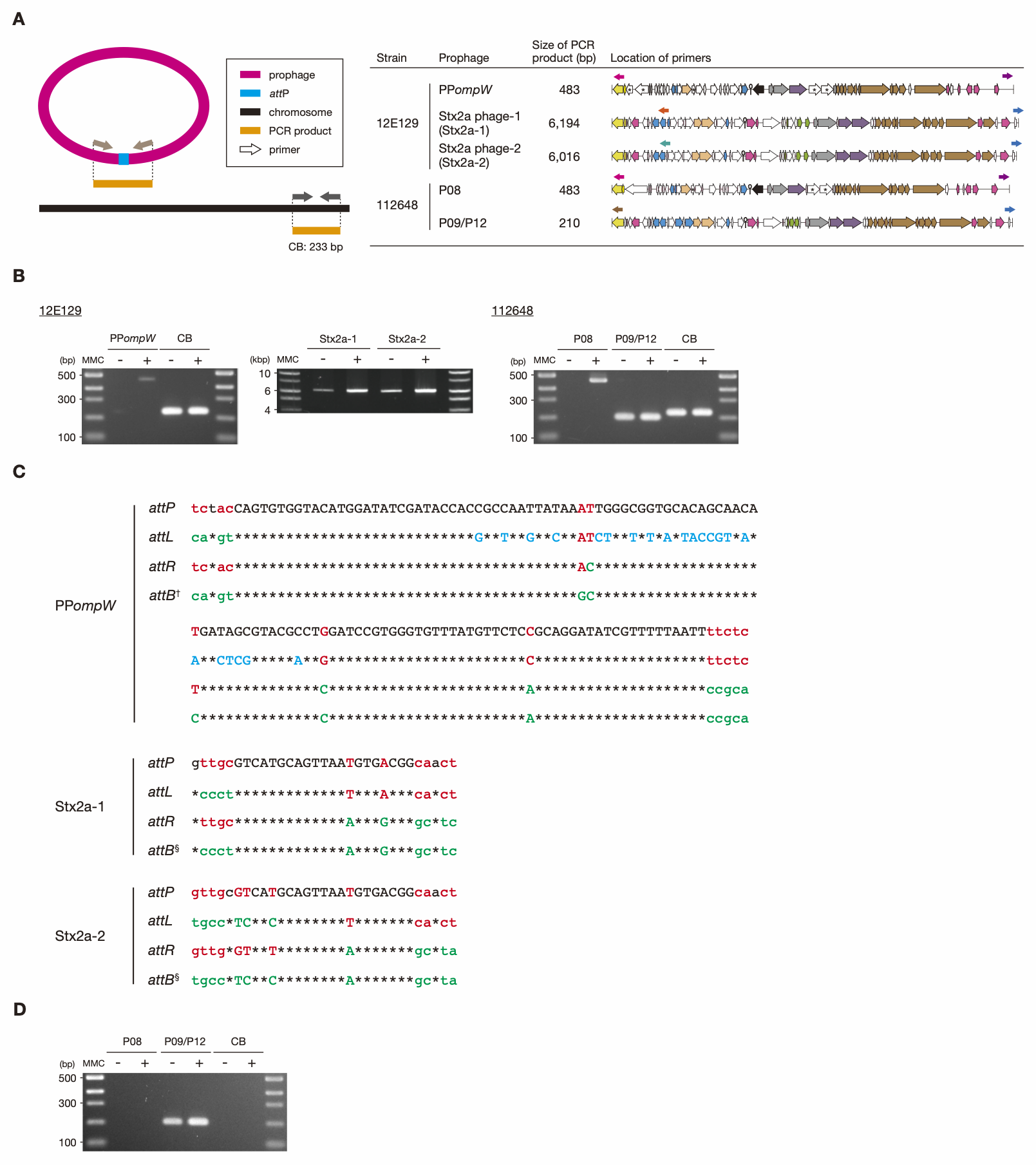

Supplement: S1 Fig — (A) Schematic representation of the PCR strategy used to amplify the attP-flanking region (left panel) and the locations of PCR primers used for each phage (right panel). (B) PCR detection of excised and circularized prophage genomes. Total cellular DNA isolated from MMC-treated (+) or MMC-untreated (-) cells was analyzed. A chromosome backbone (CB) region was amplified as a positive control. (C) The att sequences of the three prophages in strain 12E129. The attP-containing sequences obtained by sequencing the PCR products shown in S1B Fig were aligned with the attR-, attL-, attB-containing sequences to define the att sequences of each phage. The ompW sequences of strain K-12 MG1655 (accession No. NC_000913) and the yecE sequence of O145:H28 strain 122715 (accession No. AP019708), in which no phages were integrated, were used as the attB sequences, respectively (indicated by a dagger and a section mark, respectively). The defined att sequences are indicated by uppercase letters. (D) Detection of packaged DNA of the three prophages in the DNase-treated lysates of strain 112648 with (+) or without (-) MMC treatment. The CB region was amplified as a negative control. The same primer pairs as shown in S1A Fig were used in this analysis. (TIF) [file ppat.1009073.s001.tif]

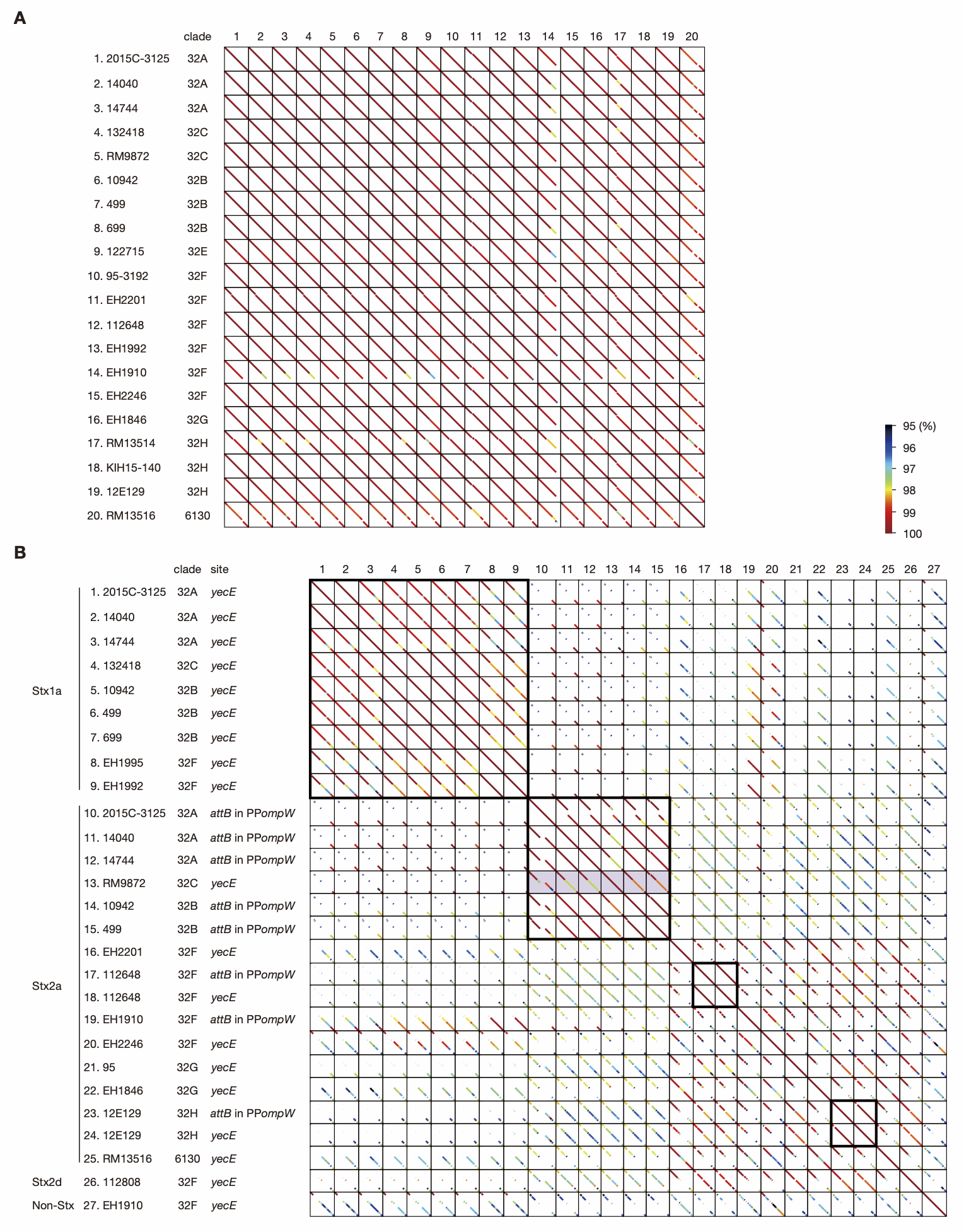

Supplement: S2 Fig — Dot plot matrixes of the concatenated sequences of the 20 PPompW genomes (A) and 27 prophage in the attB in PPompW and PPyecE genomes (B) found in 64 O145:H28 strains are shown. Strain names and information on the ST and ST32 clade of each strain are indicated. Sequence identities are indicated by a heatmap. In panel A, the nucleotide sequences between the attB in PPompW and the attR (approximately 428 bp in length) were excluded from this analysis because the sequences of this region in three strains (499, EH1910, and KIH15-140) were not determined. Average nucleotide identities (ANIs) among these genomes ranged from 97.0% to 99.9%. In panel B, the subtype of Stx encoded by each prophage and the integration site of each phage are indicated. Prophage groups sharing similar genomic sequences are framed by boxes. ANIs among the Stx1a phages ranged from 98.1% to 99.9% and those between Stx2a phage of strain RM9872 and the other Stx2a phages of clade A/B strains framed by purple boxes showed over 98.1%. (TIF) [file ppat.1009073.s002.tif]

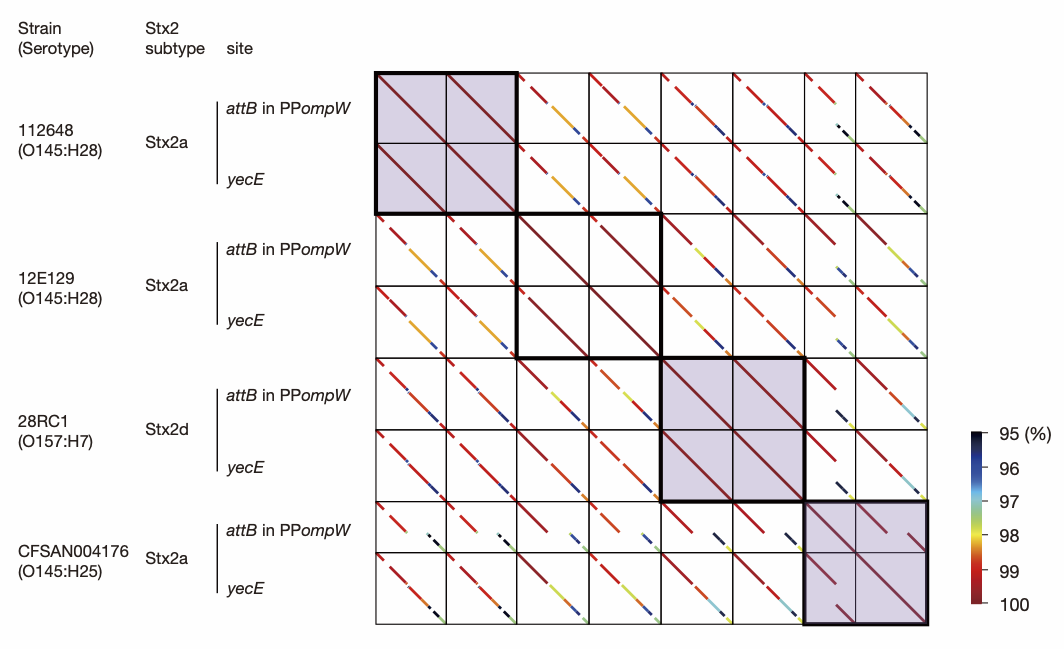

Supplement: S3 Fig — Dot plot matrixes of the concatenated sequences of the Stx phages found in four strains (two O145:H28 strains, an O157:H7 strain, and an O145:H25 strain) in their attB in PPompW and yecE loci are shown. The names of host strains, Stx2 subtypes, and integration sites of each Stx2 phage are indicated. Stx2 phages in the same strain are framed by boxes. The two Stx2 phages in three strains (indicated by purple boxes) showed high sequence identity across their entire genomes (ANI: >99.8%), suggesting that they were duplicated in each strain. Sequence identities are indicated by a heatmap. (TIF) [file ppat.1009073.s003.tif]

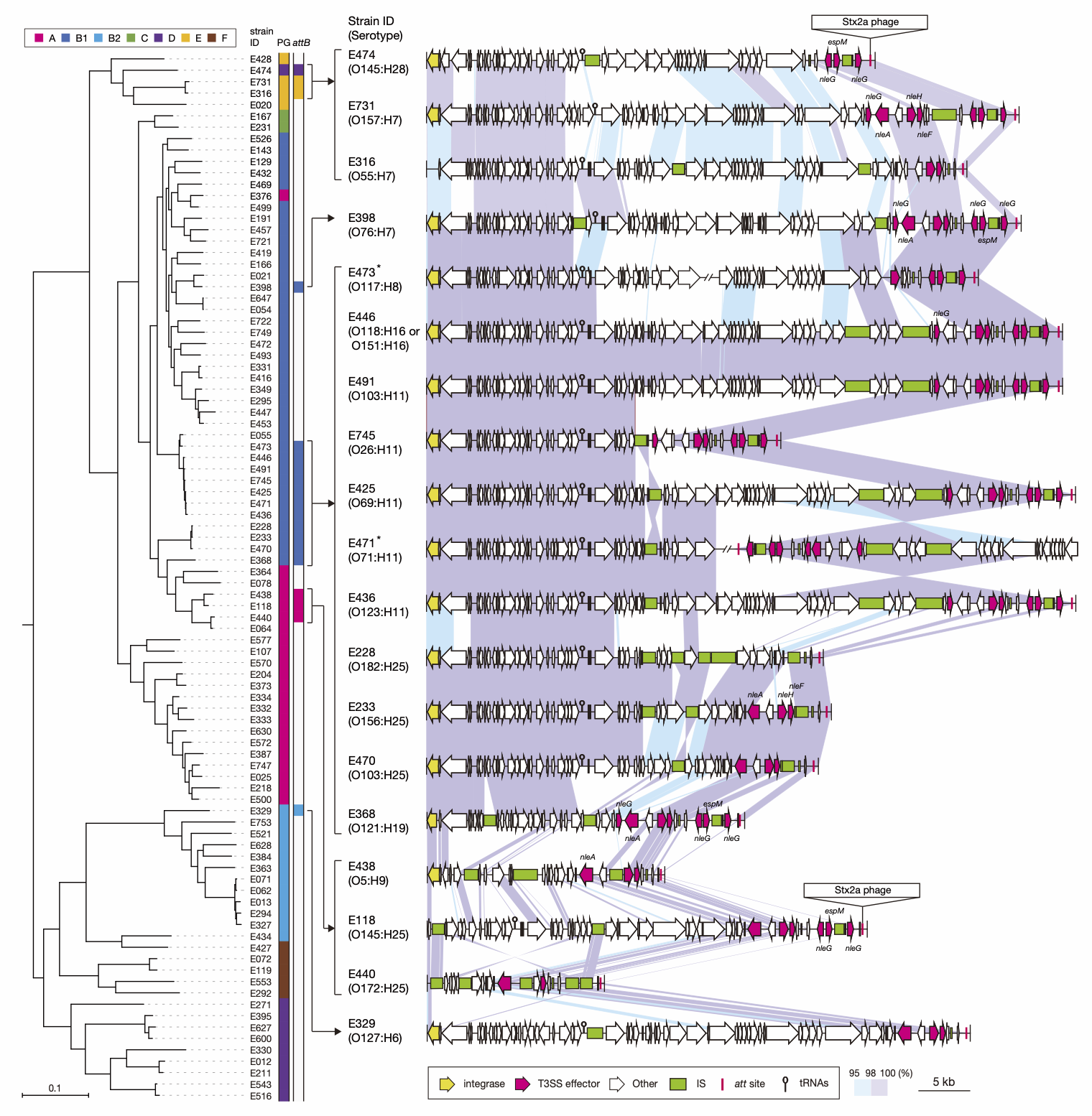

Supplement: S4 Fig — In the left panel, along with the same ML tree as shown in Fig 3, E. coli strain ID, phylogroup (PG), and the presence (colored) or absence (open) of the 21-bp attB sequence in each E. coli are indicated. In the right panel, the genome structures of PPompWs containing the attB are drawn to scale. In two strains indicated by asterisks (E473 and E471), recombination between PPompW and another prophage along with translocation of chromosome segments caused complicated chromosome inversions around the replication terminal; therefore, only relevant prophage regions are shown. Homologous regions and sequence identities are depicted by shading with a color gradient. The Stx2a phages integrated into the attB locus in strains E474 and E118 are schematically indicated. (TIF) [file ppat.1009073.s004.tif]

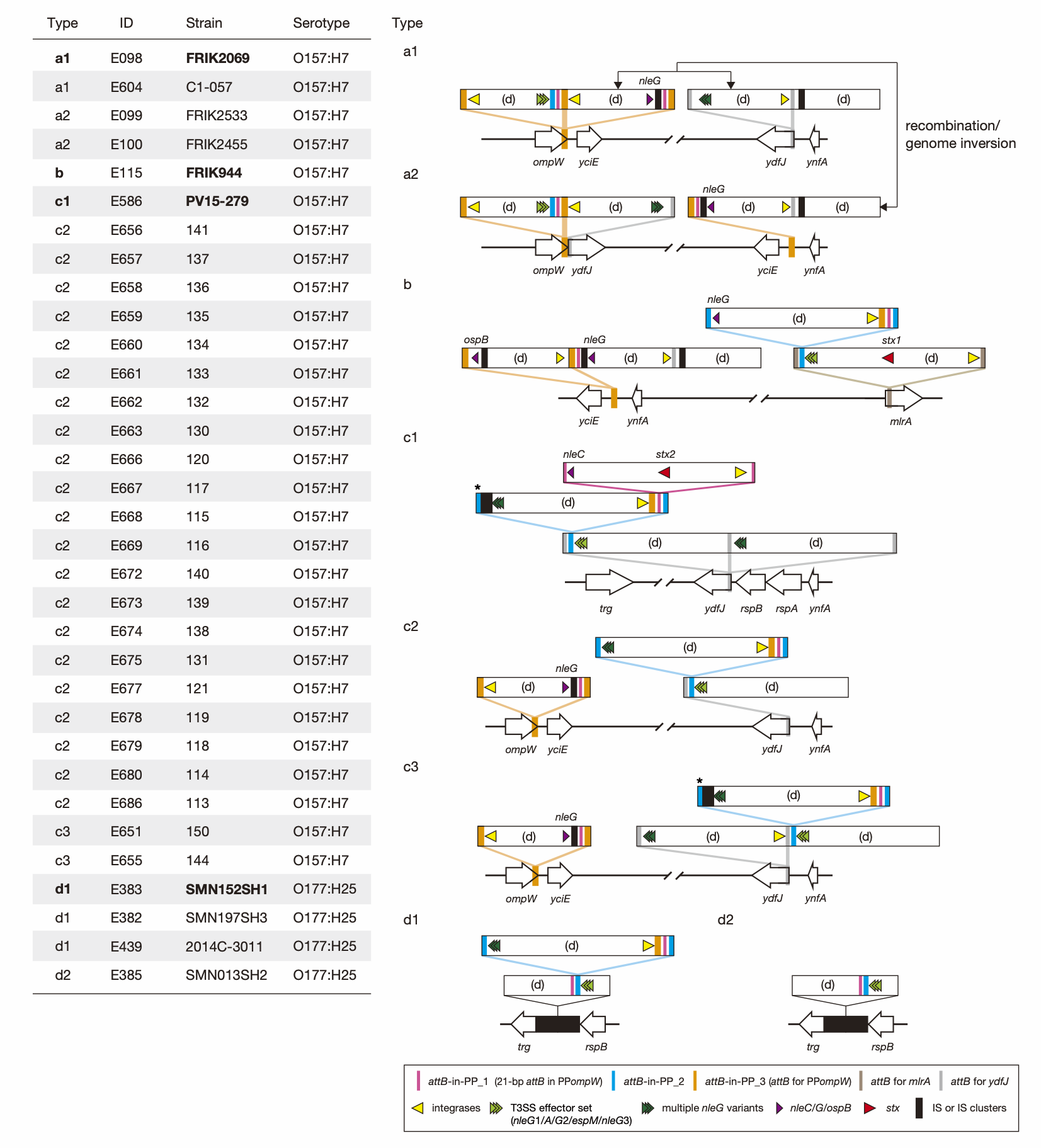

Supplement: S5 Fig — In the left table, a list of 33 strains that possessed prophage clusters that contained prophages carrying the 21-bp sequence identical or nearly identical to that of the attB in PPompW is provided. In the right panel, the patterns of phage integration are schematically illustrated. Strains showing each pattern are also indicated in the left table. CDSs shown by colored triangles include pseudogenes. The 21-bp sequence (renamed attB-in-PP_1) and other attB sequences are indicated. Among these sequences, the two indicated by an asterisk are truncated by IS insertion. Several attB sequences are missing because of deletions. The T3SS effector set (light green triangles) consists of any of the seven effector family/subfamily genes that are encoded by the PPompW EELs shown in Fig 3. Prophage that are apparently defective due to multiple gene degradation and deletion are indicated by (d). Genomic structures of four prophage clusters (indicated in bold in the left table) are presented in Fig 4. Types a, c, and d include a minor variation; homologous recombination between the second PPompW and the first PPydfJ (type a2), integrase-deficient PPydfJs with or without additional phage integration in tandem (types c2 or c3, respectively), and a region comprising two degraded prophages integrated in tandem between the trg and rspB genes without phage integration into the attB-in-PP_2 locus (type d2) are shown. (TIF) [file ppat.1009073.s005.tif]

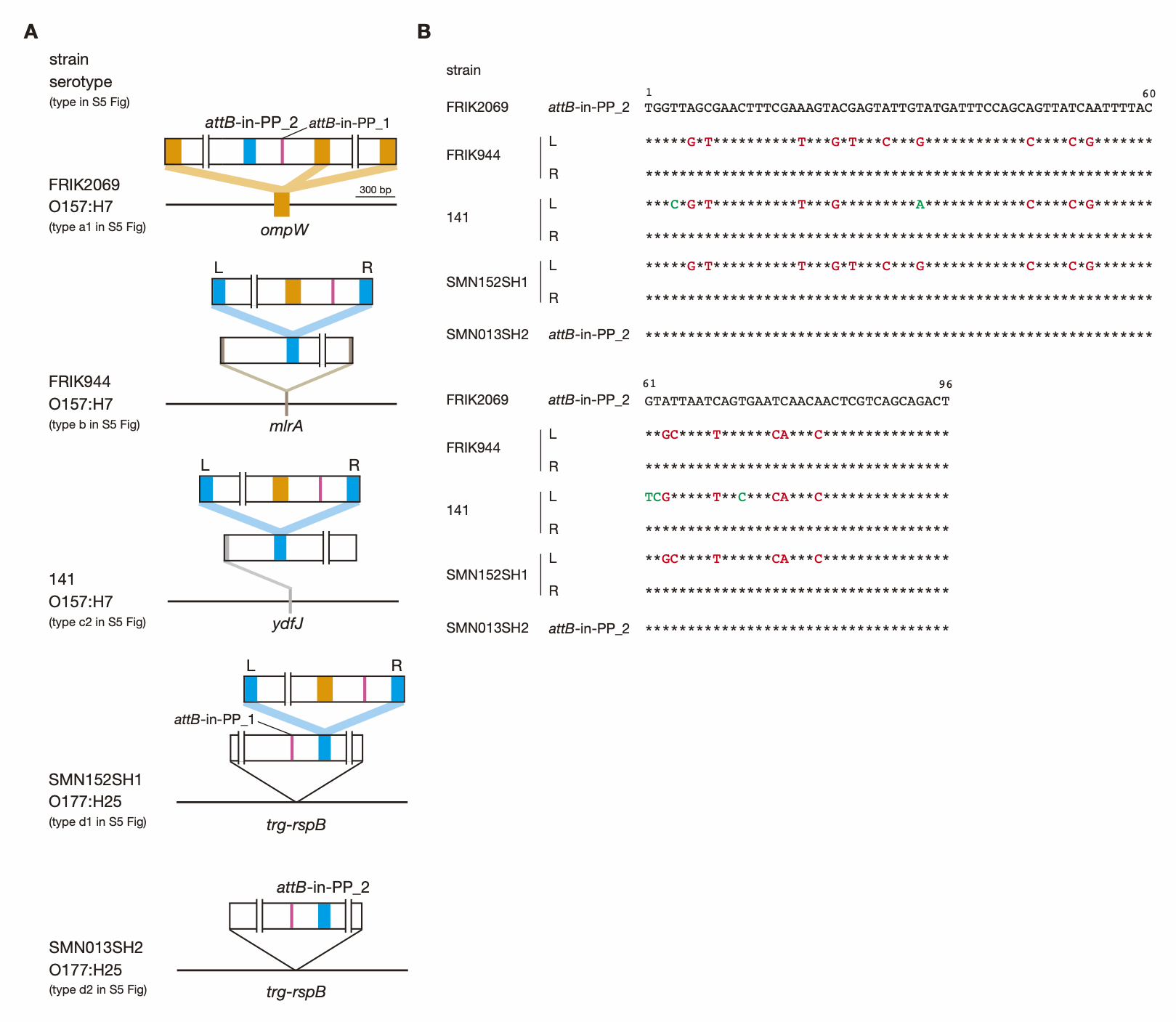

Supplement: S6 Fig — (A) Locations of the attB-in-PP_2 sequences in representative prophage genomes. (B) Comparison of the nucleotide sequence of attB-in-PP_2 among the prophages shown in panel A. (TIF) [file ppat.1009073.s006.tif]

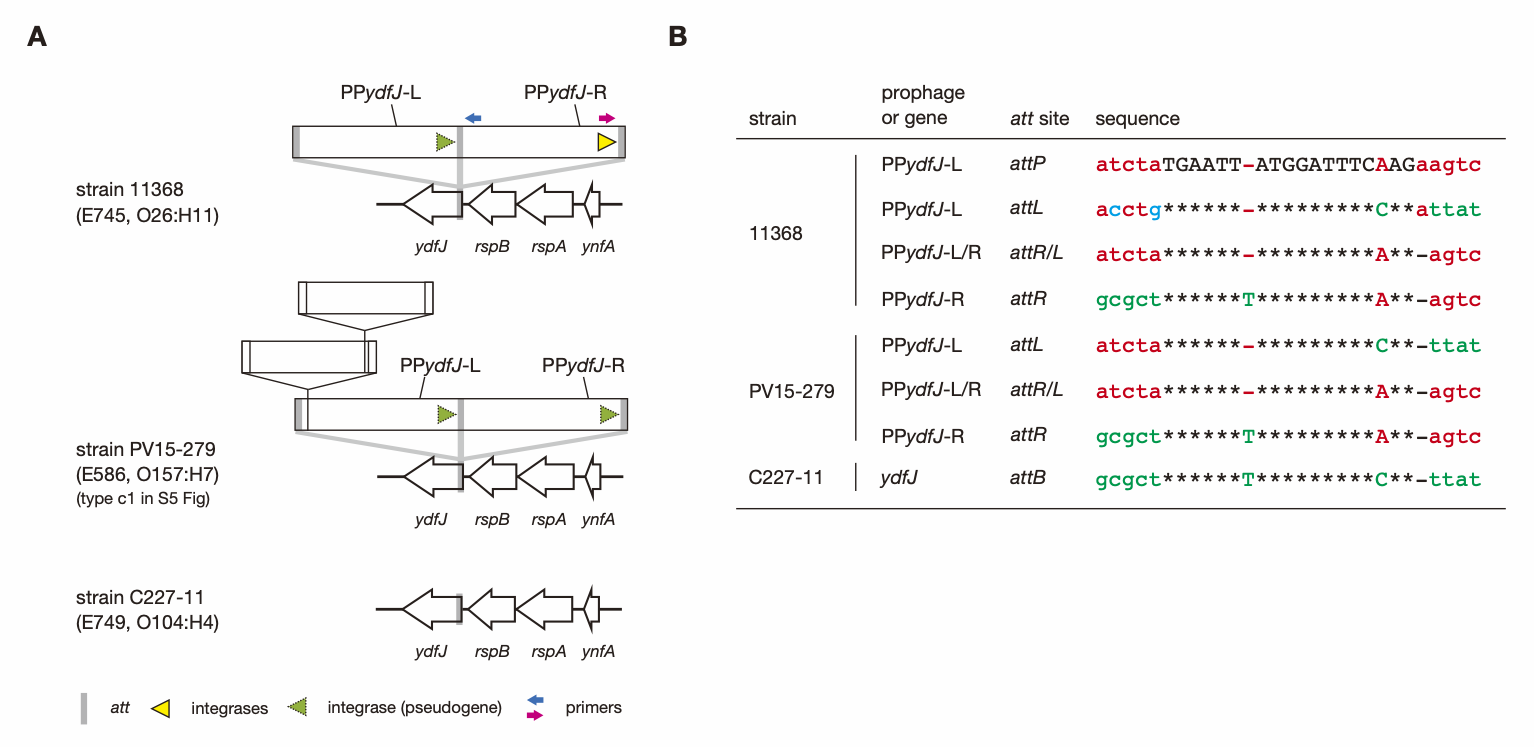

Supplement: S7 Fig — (A) Schematic representation of the ydfJ-flanking region and the prophage clusters present at the ydfJ locus in three E. coli strains. Because the integrase genes of the PPydfJs in strain PV15-279 (PPydfJ-L and PPydfJ-R) have both been inactivated by IS insertion, the PPydfJ-R of O26:H11 strain 11368 was used for sequence determination of the attP-flanking region of PPydfJ by sequencing a PCR amplicon obtained with two primers (indicated by red and blue arrows). (B) The att sequences of the four PPydfJs. The attP-containing sequence of the PPydfJ-R of strain 11368 was aligned with the attR-, attL-, and attB-containing sequences to define the att sequences of each phage. Because phages are integrated in the ydfJ locus in many E. coli strains including K-12, the ydfJ sequence of O104:H4 strain C227-11, in which no phage was integrated in this locus, was used as the attB sequence. The 18- or 19-bp att sequence that we defined is indicated by uppercase letters. (TIF) [file ppat.1009073.s007.tif]

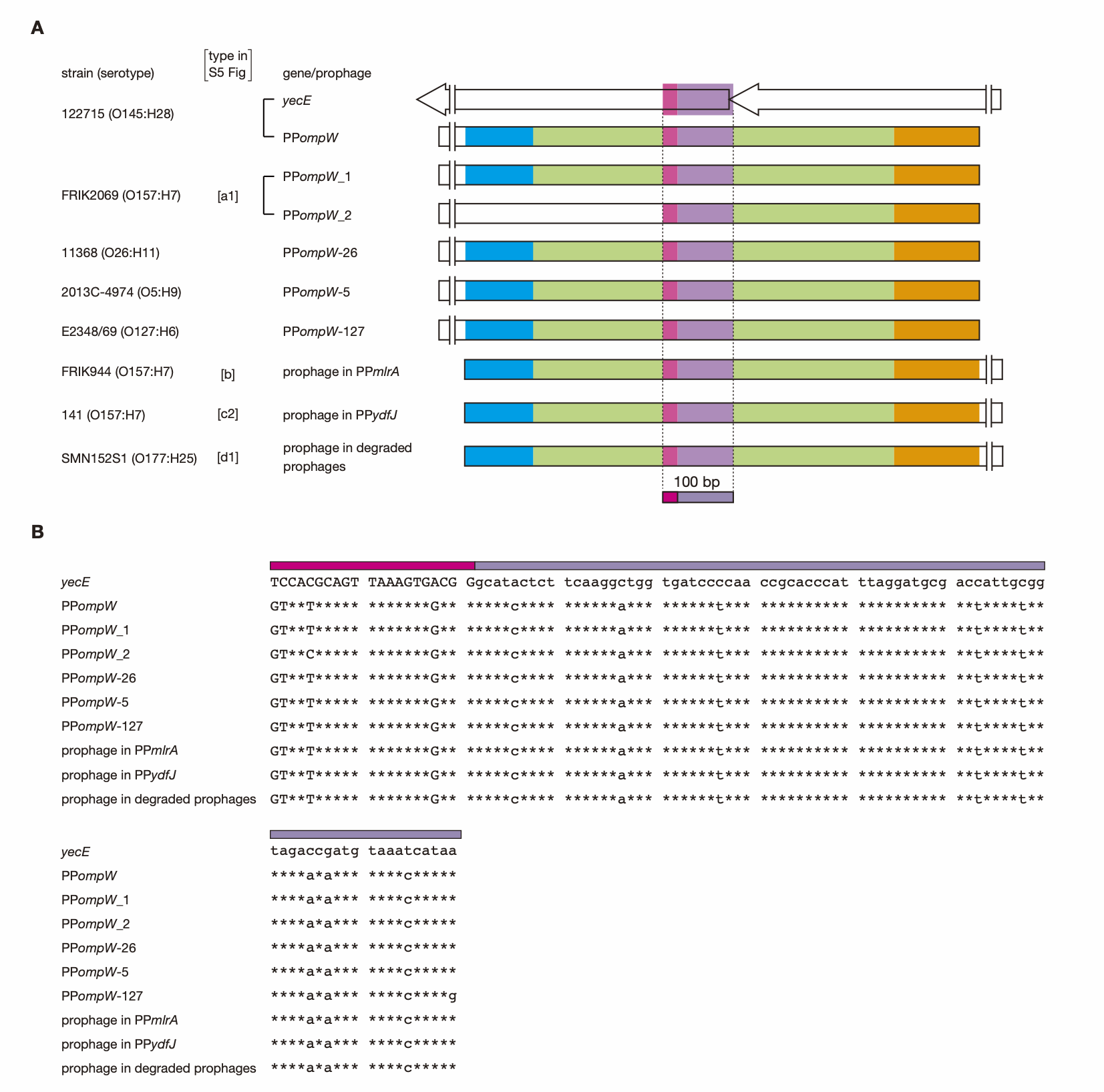

Supplement: S8 Fig — (A) The locations of the attB-in-PP_1 (initially called 21-bp attB in PPompW) sequences in the genomes of six PPompWs and three other phages integrated in prophages and in the yecE locus of E. coli O145:H28 strain 122715. The 21-bp attB-in-PP_1 sequence and the additional 79-bp sequence homologous to the yecE gene are indicated by red and purple, respectively. The attB-in-PP_2 and attB-in-PP_3 are also indicated by blue and orange, respectively. The sequences of the two regions indicated by green are conserved between prophages with up to 5 SNPs. The lengths of the two regions are 185 bp (left) and 228 bp (right). (B) Alignment of the 100-bp sequences homologous to the yecE locus in the nine prophages shown in panel A with the corresponding sequence of the yecE locus of strain E. coli O145:H28 strain 122715. The 21-bp attB-in-PP_1 sequence is indicated by uppercase letters. The 100-bp sequences of these prophages were 87% identical to the yecE sequence. (TIF) [file ppat.1009073.s008.tif]

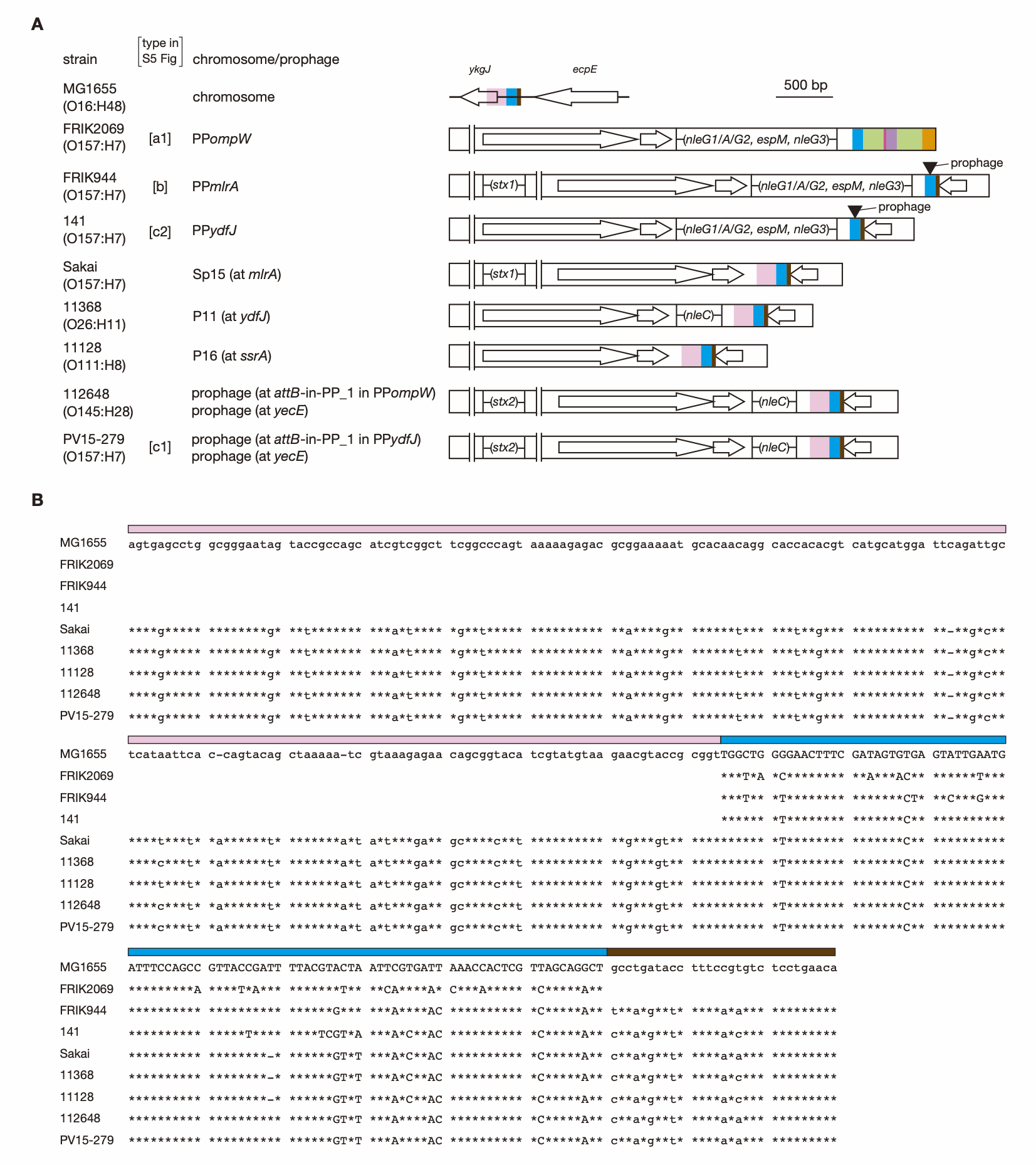

Supplement: S9 Fig — (A) The locations of the attB-in-PP_2 sequences (blue) in eight prophage genomes and on the chromosome of E. coli K-12 strain MG1655. The 96-bp attB-in-PP_2 sequences and their flanking sequences (184 bp and 29 bp in length) homologous to the ykgJ-ecpE region on the E. coli MG1655 chromosome are indicated by blue, pink, and dark brown, respectively. The presence of stx and T3SS effector genes in each prophage is also indicated. (B) Alignment of the attB-in-PP_2 and its flanking sequences in the prophages shown in panel A with the corresponding sequence of the ykgJ-ecpE region on the E. coli MG1655 chromosome. Only the prophage genomic regions homologous to the ykgJ-ecpE region are shown. The 184-bp regions (pink) of prophages show 83% sequence identity with the ykgJ-ecpE region. Note that the 96-bp attB-in-PP_2 (blue; indicated by uppercase letters) contained 23 SNPs. (TIF) [file ppat.1009073.s009.tif]

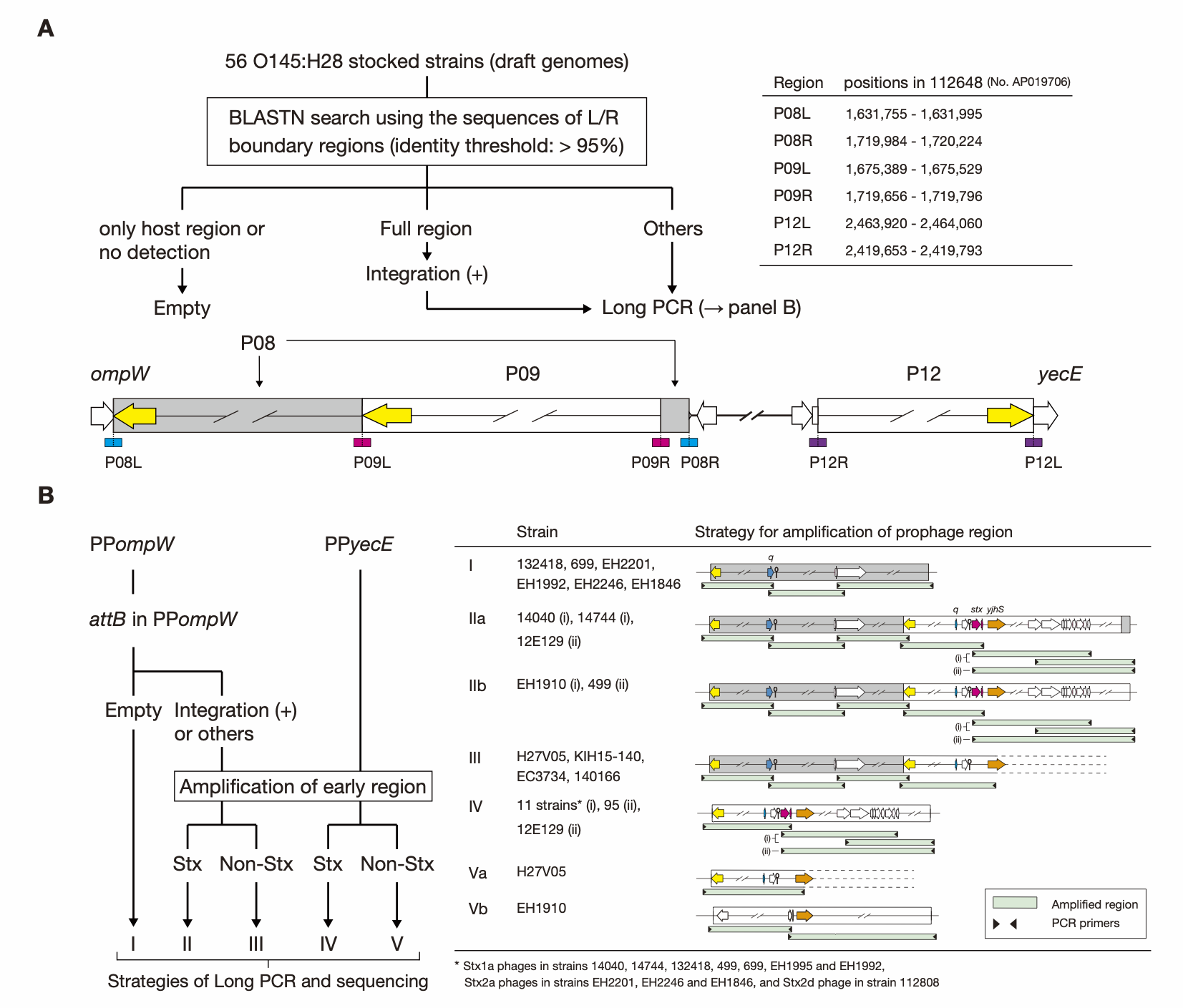

Supplement: S10 Fig — (A) Analysis of phage integration by a BLASTN search. Draft genomes of O145:H28 (n = 56) were searched by BLASTN, using the sequences of the attL- and attR-containing regions of the prophages at ompW, attB in PPompW and yecE in strain 112648 (P08L/R, P09L/R, and P12L/R, respectively) as queries. Each query sequence was composed of the sequences from the host chromosome and prophage (60 bp each) with the att sequence determined in this study (121 bp for P08 and 21 bp for P09/P12) located between them. Phage integration at each locus was considered positive when attL- and attR-containing sequences were both detected (identity threshold: >95%). Phage integration in all but two genomes was determined by this analysis. In strains EH1910 and H27V05, although phages integrated into yecE (PPyecE) were detected, PPompW was not detected. Unexpectedly, however, the P09L/R sequences (corresponding to the attL- and attR-containing sequences of the prophage in PPompW) were detected in EH1910, and a partial P09 attL sequence (74.5% coverage) was detected in H27V05. Therefore, the ompW and attB in PPompW loci of the two genomes were defined as ‘Others’, and subjected to long PCR analysis along with the identified prophages. (B) Long PCR analysis and sequence determination of prophage genomes. Strategies for five types of analysis are shown. Type I analysis: The genomes of PPompWs that did not contain prophages were divided into three segments and amplified by three long PCRs to obtain the PCR products for genomic sequence determination. Note that the left and right segments included the left and right PPompW-chromosome junctions, respectively (the same strategy was employed in Types II-V analyses). Type II analysis: The genomes of PPompWs that contained an Stx phage were amplified together with the Stx phage genomes using 5 or 6 primer pairs to confirm the presence of these prophages and to obtain the PCR products for genome sequence determination. Two primers targeted the stx gene [file ppat.1009073.s010.tif]
