# Supplementary material for: Prophages integrating into prophages: A mechanism to accumulate type III secretion effector genes and duplicate Shiga toxin-encoding prophages in Escherichia coli
Source: PLoS Pathog. 2021 Apr 29;17(4):e1009073. doi: 10.1371/journal.ppat.1009073 (PMC8112680; doi:10.1371/journal.ppat.1009073)
Supplement: Attachment — Submitted filename: Response_to_Reviewers_PPATHOGENS-D-20-02305.docx [file ppat.1009073.s018.docx]

RE: “Prophages integrating into prophages: a mechanism to accumulate type III secretion effector genes and duplicate Shiga toxin-encoding prophages in *Escherichia coli*.” By K. Nakamura *et al.* (Ms. No. PPATHOGENS-D-20-02305)

**Responses to the Reviewers**

**Reviewer 1:**

**Part I - Summary**

Nakamura et al report an impressive, elegant and very precise analysis of a new observation, namely the faculty for a prophage to host another prophage inside its genome, which they name PP in PP for short. They detail this observation starting from a set of STEC strains of E. coli, but then extend this analysis across all E. coli, and end up suggesting that it may be an even more frequent phenomenon in nature.

<Response>

Thank you for encouraging comments and many thoughtful and valuable suggestions, all of which were helpful for us to improve our manuscript. Please note that line numbers indicated in this Responses correspond to those in the revised manuscript. Please also see the attached word file in track changes mode.

**Part II - Major Issues: Key Experiments Required for Acceptance**

I have very little questions/suggestions to improve this already very complete work.

1. From Figure 1c one gathers that the lambda in ompW is defective in strain 12E129. It has indeed an IS in the structural region. Do you have elements to suggest that this is always the case when the PPompW prophage is hosting a PP-in-PP? Do they all have IS for instance? More broadly, are all PPompW predicted defective, regardless of their “occupation” (44% of E. coli strains having PPompW seems a lot…)?

<Response>

The reviewer is correct, and we overlooked this point. We re-analyzed the genomes of the lambda-like phages in *ompW* in O145:H28 strains 112648 and 12E129 and found that, in addition to an IS-associated deletion of the lysis gene cassette-encoding region, a gene for head formation (portal protein-homologue) has been inactivated in the two phages. Both phages contained one or more additional pseudogenes. We further re-analyzed all PP*ompW*s (prophages in *ompW*) analyzed in this study (those in the 20 O145:H28 strains shown in S2A Fig, the 20 *E. coli* strains shown in S4 Fig, and the 28 O157:H7 strains shown in the S5 Fig), and confirmed that all contained multiple gene degradations and deletions. We also confirmed that no packaged DNA can be detected for the PP*ompW* of strain E2348/69, which contained a relatively well conserved gene set (E329 in Fig 3 and S4 Fig). Thus, as pointed out by the reviewer, they are apparently defective. These findings have been described in the revised text (lines 114-117, lines 134-136, lines 190-193, and lines 257-260). In addition, all prophages being integrated by another phage, which we found in other sites (S5 Fig), were also found to be apparently defective (this information has been added to the revise text; lines 257-260). Thus, *attB* sites in defective prophages may be favorable sites for phage integration. This possibility and its biological significance were briefly mentioned in the revised Discussion (lines 312-315).

2. The fact that strain 12E129 hosts two copies of prophage Stx2a suggests that immunity to super-infection did not “work”. Or was it a single infection followed by two simultaneous integration events?

<Response>

This point is also what we overlooked. As indicated in the revised Fig. 1, the *cI*-encoding regions were different in sequence between the two Stx2a phages and the leftmost regions showed significantly lower sequence identity (98.8% identity) compared to other parts of the genomes which were identical. Thus, at least two different scenarios can be considered for the acquisition of two copies of Stx2a phage in strain 12E129; segment replacement after phage duplication or independent infection (super-infection of the second copy). These possibilities have been described in the revised text (lines 96-98). Please note that, in two non-O145:H28 strains which also contained two copies of Stx2a phages (S3 Fig), two copies were nearly identical (>99.8% sequence identity across the entire genome) although a large deletion occurred in one strain. Thus, in these strains, duplication of Stx2a phage similar to that in the O145:H28 strain 112648 appears to have occurred. This point was also briefly described in the revised text (lines 180-182), and S3 Fig and its legend (lines 613-615) have been modified to clarify it.

Are the two copies in direct or inverse orientation on the chromosome of E. coli?

<Response>

The two Stx2a phages were integrated in an inverse orientation in respect to the *ori*-*ter* axis. Please see the reviewer-only-figure (the last page in this file) which shows a circular map of O145:H28 strain 112648 and the positions and directions of prophages including two Stx2a phages. The circular map of K12 strain MG1655 is also shown in this figure.

3. From Fig2, it seems that the PP-in-PP event occurs only when the first att site (yecE) is occupied. Why should it be so?

<Response>

We also considered a possibility that the *attB* in PP*ompW* represents a secondary attachment site because the *yecE* site has been occupied in many of the O145:H28 strains that carried a prophage in PP*ompW*. However, there were three strains that contained no prophage at *yecE* but carried a prophage in PP*ompW* (gray triangles in Fig 2), while the *yecE* genes of these strains were intact. Therefore, although we also prefer this possibility (the *attB* in *yecE* is a more preferable integration site), we have decided not to mention it in this manuscript. The preference of *attB* sites by Stx phages is an important target of our next project.

**Part III – Minor Issues: Editorial and Data Presentation Modifications**

4. Line 67: ”locus-encoding”, remove locus-

<Response>

Thank you for pointing this out. We have deleted “locus-” (line 67)

5. Line 72: it was hard at the onset to understand precisely what the duplication meant. I propose some rewriting such as :”we initially analyzed the two copies of an Stx2-encoding PP present in two loci of a STEC O145:H128 genome, …”

<Response>

Following the reviewer’s suggestion, we have modified this sentence (lines 72-73).

6. Lines 84-88: along the same line, to help the reader enter into the PP jungle, some suggestions: “As the lambda-like P08 prophage was also found at ompW”. The next sentence begining with “However, by analyzing” is really hard to follow, and important for the following. Avoid the “respectively” formula and may be, cut the sentence. This onset of the work has to be crystal clear. I had to draw a little cartoon for myself to make it clear.

<Response>

Following the reviewer’s suggestion, we have modified or reconstructed these sentences (lines 88-90).

7. Line 152: "by filtering the att sequence in yecE", you mean you masked it?

<Response>

In this analysis, we searched for 21-bp sequences by BLASTN, and then removed the 21-bp sequences in *yecE* gene from the data set (please see the Material and Methods; lines 399-400). To make it clearer, we have changed this phrase to "after excluding the 21-bp *attB* sequence in *yecE*" (lines 164-165).

8. Line 162: I am a bit lost here, about the 145 strains found in 4 different phylotypes: do you mean that these strains all belong to serotype O145:H28 except 3?

<Response>

The 150 strains that contained non-*yecE* 21-bp sequences were found in five phylogroups. Among the 150 strains, 145 contained the 21-bp sequence in PP*ompW*, but 28 of the 145 strains (all were O157) contained an additional 21-bp sequence. The remaining five strains contained one or two 21-bp sequences in a prophage or prophage cluster at *ydfJ*. As this point was not clear in our original text, we have fully revised this part to make it clearer (lines 164-173).

**Reviewer 2:**

**Part I - Summary**

Compound prophage regions are a feature of some STEC genomes especially near the terminus of O157 strains. The prophages that are present and recombination between them underpins the majority of structural variation in these strains. New incoming phages with lysogenic potential can only integrate if there is an available attB (integration site in the bacterial xome) and certainly for the majority of Shiga toxin-encoding prophages which are generally Lambda-like, then these sites have been very well characterized including wrbA, yehV, sbcB and yecE.

The manuscript by Nakamura et al is primarily descriptive and based on genome analysis initially of STEC O145:H28 isolates followed by casting the net wider to other STEC and more general E. coli. Its key observation is that Stx2a-encoding and other prophages can insert into an ‘ompW’ attB site near the end of a prophage already inserted into the ‘original’ ompW-attB site. In this way they state that compound phages are produced by integration of one phage into another. They surmise that the PP-attB site is the likely integration site by showing that the Stx2a prophages can excise cleanly from this site. An important observation of this study though is that the original PP-ompW is fixed and does not excise as a functional phage, perhaps not surprisingly given the integrated phage within it.

<Response>

Thank you for many thoughtful comments on our manuscript. Although it was difficult for us to address some comments, we have tried our best. Please note that line numbers indicated in this Responses correspond to those in the revised manuscript. Please also see the attached word file in track changes mode.

The functionality of prophages in *ompW*, which was also pointed out by other reviewer, was what we missed in the original manuscript. We have re-analyzed the genomes of all prophages in *ompW* (PP*ompW*s) analyzed in this study (those in the O145:H28 strains shown in Fig. 1 and S2A Fig and other *E. coli* strains shown in S4 and S5 Figs), and found that all PP*ompW*s contained multiple gene degradations and deletions. We also confirmed that no packaged DNA can be detected for the PP*ompW* of strain E2348/69, which contained a relatively well conserved gene set (E329 in Fig 3 and S4 Fig). Thus, as pointed out by the reviewer, they are apparently defective. These findings have been described in the revised text (lines 114-117, lines 134-136, lines 190-193, and lines 257-260). In addition, all prophages being integrated by another phage, which we found in other sites (S5 Fig), were also found to be apparently defective (this information has been added to the revise text; lines 257-260). Thus, *attB* sites in defective prophages may be favorable sites for phage integration. This possibility and its biological significance were briefly mentioned in the revised Discussion (lines 312-315).

The study demonstrates that there is a more or less conserved prophage at this site in this serotype and that has this prophage attB site and so can presumably receive prophages. The critical thing from my point of view for the overall significance of the manuscript is how general is such acquisition, and therefore compound phages built this way, and the process by which an integrated prophage would ‘acquire’ such an attB site. As suggested by the authors the most likely is duplication and recombination but this process is unknown at this point. Intra-chromosomal recombination events are common in these strains, enabled by multiple similar prophage regions as well as IS elements that promote homologous recombination. From one perspective it is not surprising that an attB site could be duplicated as part of a large duplication potentially with inversion and deletion events. It is also possible that the Stx2a-encoding phage is actually copied as part of this process without necessarily being inserted as a separate event. A ‘chicken and egg, argument. Linked to this I think they are missing a trick by not including a wider chromosome structure to prove its phages moving from yecE to the att inside the phage at ompW and while less likely that it’s a large structural variant this is a possibility as the prophages at ompW/RspR/mlrA are almost always compounded because of large-scale genome recombination events.

<Response>

We agree that intra-chromosomal recombination events between prophages might be involved in the acquisition of *attB* sequences by prophages, and such recombination, particularly those inducing symmetric chromosome inversions in respect to the *ori*-*ter* axis, contributed to the formation of complex prophage clusters and spread of intra-prophage *attB* sequences. These possibilities were mentioned in the revised Discussion (lines 317-321). In addition, we modified seven figures (Figs. 2, 4, 5 and S4, S5, S6, S7 Figs) so that readers can identify such recombination events found between prophages/prophage clusters and more easily understand the relative locations of *attB*-containing prophages/prophage clusters. However, please note that the *ykgJ-ecpE* region (the putative origin of the *attB*-in-PP_2) is not located around the *ter* region (please see the Reviewer-only-figure attached to the last page in this file) showing a circular chromosome map of O145:H28 strain 112648 and the positions and directions of prophages in this strain, as well as the circular map of K12 strain MG1655). This indicates that the mechanism/process behind the acquisition and spread of this *attB* sequence is not so simple. As for the possibility that "the Stx2a-encoding phage is actually copied as part of this process without necessarily being inserted as a separate event", this possibility seems unlikely because we could not find any sign of recombination/inversion between the two copies of Stx2 phages in any strains that were found to contain two Stx2 phages in this study.

**Part II - Major Issues: Key Experiments Required for Acceptance**

The authors analyse very different serotypes in the end, which I understand are there to see how broad the concept they are proposing might be. But the way it is presented actually makes the work harder to follow as the sites described soon spiral out to related putative attB sequences with no functional analysis.

The work is much tighter and easier to follow focused on O145. Conceptually, active phage cannot really have their own attB site within them as the moment they integrate they would become a target for prophage insertion and so would ‘kill themselves off’, so the event here is one outcome of chromosomal rearrangements involving prophage that are important for generating strain diversity.

<Response>

We agree that if the study focused on O145:H28, it would be easier for readers to follow the manuscript. However, we believe that, to show the generality of our findings (the wide distribution of phages integrating into prophages and their association with T3SS effector loci in various *E. coli* serotypes/lineages, not in limited ones), the current results analyzing all publicly available complete genomes need to be presented. However, as the original manuscript contained many descriptions that were difficult to follow, we have improved the readability through the entire manuscript as much as possible by changing terms/phrases (such as "PP"; please see our response to the last comment from the reviewer) and modifying figures and tables.

Regarding the non-functionality of "host" prophages, we have clarified it (all were apparently defective) as explained in our response to the Summary comment. As pointed out, this study lacks functional analyses of putative second and third *attB* sequences in prophages (derived from the *ykgJ*/*ecpE* intergenic region and the *ompW* gene). This was also related to the non-functionality of the prophages integrating into these *attB* sequences. As indicated in the revised S5 Fig, all prophages found to integrate into the second *attB* contained multiple defects. Thus, we could not perform functional analyses of this *attB*. As for the third *attB*, it would be interesting if we could show experimentally they can actually function as the *attB* sequence, but unfortunately all prophages integrated into *ompW* were defective as explained above.

We also agree with the reviewer's comment on that the involvement of chromosome rearrangements as described in our response to the Summary comment.

While the compound phage origins and associated genomics are intriguing and coherent, I am less convinced by the argument then used in relation to acquisition of putative T3SS effectors. This seems to be a focus more to fit in with a pathogenesis argument when the attB PP in PP concept which is more about general phage evolution. Yes, STEC strains with a LEE locus have acquired effectors over time, the majority encoded on prophages. The additional attB site does allow for an extra inserted/duplicated phage but there is no evidence provided that this organization can facilitate linked effector expression or if there is any other advantage over integration at any other site. I also found it difficult to work out from the supplementary data if the effectors were restricted to eae+ strains, i.e. were only/mainly present in strains with a T3SS.

<Response>

As pointed out by the reviewer, we provide no evidence to suggest that the association of *attB* and T3SS effectors facilitate expression of these effectors and confer any advantages to host bacteria. We only showed this association promoted the accumulation and variation of effector genes in EPEC and typical STEC strains. Unfortunately, it is very difficult at moment to analyze how these newly acquired T3SS effector genes affect the virulence potentials of the host strains. Therefore, we removed the comment on a possibility that it may increase the pathogenicity of these strains, and instead added a comment "how it affects the virulence potential of these strains is currently unknown and requires further analyses" to the revised Discussion (lines 327-328) and a phrase "the evolution of EPEC and STEC and their pathogenicity" to "the evolution of EPEC and STEC and their genetic diversity" (lines 334-335).

Regarding the distribution of effectors and its association with *eae*+ strains, we have showed the presence/absence of *eae* gene in all *E. coli* complete genomes and all T3SS effector genes encoded by the phages integrating in prophages in the revised supplementary table (S5 Table, which correspond to S4 Table in the original manuscript). In addition, we modified Figs. 2 and 6, S5 Fig, S3 Table (S1 Table in the revised manuscript) to provide more detailed and precise information on T3SS effectors in each prophage, and added a summary comment about T3SS effectors in prophages of all 150 strains that were found to contain the 21-bp *attB* sequence (lines 210-212).

I think they have shown that such attB duplication/evolution allows more prophage based information to be held in the genome but the origin of such sequences and any advantage in relation to the T3S effector profile for any specific strain/serotype is not supported experimentally, so there is a fair amount of speculation.

<Response>

To be host, it is difficult to experimentally show the origin of the *attB* sequences in prophages, and we can only speculate that they were derived from the chromosomes of *E. coli* or its close relatives. We understand that this is the limitation of this study, and added a comment on this point "we were unable to address the molecular mechanism(s)" to the revised manuscript (lines 316-317). Regarding the advantage in relation to the T3S effector profile, please see our response to the previous comment from the reviewer.

**Part III – Minor Issues: Editorial and Data Presentation Modifications**

Really well produced supplementary figures, especially Fig. S4. An incredible amount of detail about the possible construction of certain composites at specific sites, but not sure about the link to the effector and impact/role on virulence or regulation.

<Response>

As described in our response to the second major comment, we were unable to show the role on virulence or regulation. So, as described in our response to the second major comment from the reviewer, we have removed the comment on a possibility that acquisition of additional effectors increases the pathogenicity of these strains, and added a comment "how it affects the virulence potential of these strains is currently unknown and requires further analyses" to the revised Discussion (lines 327-328). A phrase "the evolution of EPEC and STEC and their pathogenicity" in the last paragraph to "the evolution of EPEC and STEC and their genetic diversity" (lines 334-335). In addition, S4 Fig was modified to more precisely show the effector repertoires in PP*ompW*s.

All phage comparisons are done by BLAST and are only in text saying they are similar or not, I think it would be nice to back this up with some numbers i.e. % similarity of prophage gene content.

<Response>

Because it is difficult to show % similarity of prophage gene content between prophages, we determined average nucleotide identity (ANI) between prophages to quantitatively show the entire sequence similarity between prophages. When we needed to show the similarity of prophage, we used the ANI values throughout the manuscript in the legends of S2 and S3 Figs.

If they could show a mechanism behind such attB duplication or transposition to argue that this was an evolved general strategy to acquire more exogenous prophage information then that would be exciting. Another issue is that such PP expansion cannot continue as it will imbalance the replichores unless centered close to the terminus (as in this case?) or matched by reciprocal events on the other side of the chromosome?

<Response>

Unfortunately, we cannot show the molecular mechanism as described in our response to the last major comment from the reviewer. So, we have added a comment "we were unable to address the molecular mechanism(s)" to the revised Discussion (lines 316-317). However, in this study, we showed the wide distribution of *attB* sequences in prophages and phage integration into these *attB* sequences. This is the reason why we want to present the current results analyzing all publicly available complete genome. Regarding the problem of replichore imbalance, all prophage-in-prophage formations we found in this study occurred at the chromosome regions close to the replication terminus (please see the Reviewer-only-figure attached to the last page of this file). We have also found several reciprocal inversions on the other side of the chromosome by recombination between prophages/prophage clusters at *ompW* and *ydfJ*. We have commented on it in the revised text (lines 321-322) and indicated these events in the revised Fig 2 and S5 Fig.

I find their nomenclature of ompW-PP and att-in-ompW-PP and stx2a-PP does make it harder to read rather than easier.

<Response>

We agree that too much use of PP makes it harder to read the manuscript. Following the reviewer’s suggestion, we have changed “PP” to “prophage”, Stx-PP to “Stx phage” and “*att*-in-PP*ompW*” to “*attB* in PP*ompW*” or “the 21-bp *attB*” in the revised manuscript (including those appeared in all figures and tables). However, please let us keep using "PPxxx (e.g. PP*ompW*)" and "*attB*-in-PP_1/2/3" as the terms to indicate specific prophages and *attB* sites.

**Reviewer 3:**

**Part I - Summary**

Strengths:

Analysing prophage regions of STEC genomes is very challenging and this study provides an approach on how to do that.

The manuscript is very well written.

The analysis is complex but the results section is well structured and easy to follow.

The analysis is novel and the extent to which prophage-prophage integration occurs is a significant finding.

The paper describes a mechanism by which E. coli can accumulate virulence determinants thus providing insight into how highly pathogenic variants of STEC evolve.

Understanding mechanisms of loss and acquisition of stx-encoding prophage is key to enabling us to monitoring for emerging threats to public health.

Weaknesses:

The authors should explain their strain selection criteria.

A wide range of STEC serotypes have been selected for the study - the advantage of this is that it shows this phenomenon is widespread but it may be difficult for readers unfamiliar with STEC to assimilate the data.

I think a clear explanation as to why this specific strain set was selected for this study would help.

<Response>

Thank you for many encouraging comments and constructive suggestions. We used all complete *E. coli* genomes publicly available (n=767) without selection except for excluding laboratory, commercial and re-sequenced strains and substrains. As pointed out by the reviewer, this set was biased to STEC/EPEC strains (n=252), particularly to STEC O157 (n=91). However, we used this set because detailed and accurate analyzes of prophage configurations and sequences were possible only in complete genomes. These points have been clarified in the revised manuscript (Lines 157-161) and prepared a new supplementary table (S2 Table) which provides the summary information on the 767 genomes about the presence or absence of *eae* and *stx* genes. In addition, we have modified two original supplementary tables (S1 and S3 Tables) to provide the detailed information on the distribution of serotypes, *eae* and *stx* genes, PP*ompW*, and the 21-bp *attB* sequence in all 150 genomes (all were EPEC or LEE-positive STEC) that contained the 21-bp *attB* sequence in prophages (S3 Table in the revised manuscript) and these information on each of the all 767 complete genomes analyzed (S5 Table in the revised manuscript).

**Part II - Major Issues: Key Experiments Required for Acceptance**

None identified

**Part III – Minor Issues: Editorial and Data Presentation Modifications**

The authors should explain their strain selection criteria.

A wide range of STEC serotypes have been selected for the study - the advantage of this is that it shows this phenomenon is widespread but it may be difficult for readers unfamiliar with STEC to assimilate the data.

I think a clear explanation as to why this specific strain set was selected for this study would help.

<Response>

Please see our response to the comment in Part I – Summary.

**Reviewer-only-figure. Circular maps of the chromosomes of O145:H28 strain 112648 and K-12 MG1655.** The inner circles show the GC skew. The outer circle in strain 112648 shows the locations and directions of prophages and prophage-like integrative elements (red arrow, Stx2a phage; orange arrow, prophages; light blue square, integrative elements) and the nucleotide sequence positions (in Mbp). In each prophage, *int* genes are located in the opposite side of arrow head. The location of the intergenic region between *ykgJ* and *ecpE* genes is also indicated. Although the chromosomes of two strains shared overall synteny, an inversion of the *dif*-containing segment has occurred, probably in strain 112648, by the recombination between PP*ttcA* and PP*ydfJ*.
